# Response to “Reconsidering the Perinatal Risks of Maternal Underweight in Japan”

**DOI:** 10.2188/jea.JE20260098

**Published:** 2026-08-05

**Authors:** Shinobu Kobayashi, Shiori Itoi, Drishti Shrestha, Naho Morisaki

**Affiliations:** 1 Department of Social Medicine, National Center for Child Health and Development, Tokyo, Japan; 2 Division of Reproductive Medicine, National Center for Child Health and Development Research Institute, Tokyo, Japan; 3 Department of Obstetrics and Gynecology, The University of Tokyo, Tokyo, Japan; 4 Graduate School of Public Health, St. Luke’s International University, Tokyo, Japan

Dear Editor,

We would like to thank Dr Jiayi Chen for the insightful and encouraging comments^1^ regarding our recent systematic review and meta-analysis, “Impact of Maternal Underweight on Infant and Maternal Outcomes in Japanese Women”.^2^ We are pleased that our study was recognized for its methodological rigor and its contribution to addressing the high prevalence of pre-pregnancy underweight in Japan.

Dr Chen raises a pertinent point regarding the importance of stratified analyses, particularly by age group or by the severity of underweight. We completely agree that such detailed analyses would provide more targeted insights for clinical interventions. However, as noted in the letter, our review was based on a synthesis of existing studies, many of which utilized secondary data. During our analysis, we encountered inherent limitations in these data sources, particularly regarding the consistency of how maternal age was recorded and categorized, which hindered our ability to perform robust subgroup analyses across all included studies.

To overcome these challenges, we believe it is essential to establish more integrated systems and infrastructure for managing individual-level data in the future. Such advancements would allow researchers to conduct more granular and detailed analyses, as Dr Chen suggested.

In conclusion, we appreciate the opportunity to reinforce the message that addressing maternal underweight is a vital component of maternal and child health strategies in Japan. We hope that this discussion further encourages the development of data-driven public health policies and preconception counseling.
